# Going Viral: The 3 Rs of Social Media Messaging during Public Health Emergencies

**DOI:** 10.1089/hs.2020.0157

**Published:** 2021-02-18

**Authors:** Bhavini Patel Murthy, Tanya Telfair LeBlanc, Sara J. Vagi, Rachel Nonkin Avchen

**Affiliations:** Bhavini Patel Murthy, MD, MPH, is a Lieutenant Commander, United States Public Health Service, and a Medical Epidemiologist; Sara J. Vagi, MS, PhD, is Commander, United States Public Health Service, and a Senior Health Scientist; Tanya Telfair LeBlanc, MS, PhD, was a Senior Health Scientist at the time of the study; and Rachel Nonkin Avchen, MS, PhD, is a Captain, United States Public Health Service, and Branch Chief; all in the Division of State and Local Readiness, Center for Preparedness and Response, Centers for Disease Control and Prevention, Atlanta, GA. Tanya Telfair LeBlanc, MS, PhD, is currently a Senior Health Scientist/Epidemiologist, National Center for Environmental Health/ Division of Environmental Health Science and Practice, Centers for Disease Control and Prevention, Chamblee, GA.

**Keywords:** Public health preparedness/response, Social media, Risk communication, Mass messaging, Emergency planning/response, Citizen engagement

## Public Health Risk Communication in a Digital Age

The rise of social media has transformed the way individuals share and consume information. Approximately two-thirds of Americans receive at least some of their news from social media channels such as Facebook, Twitter, YouTube, Instagram, and Snapchat.^1^ During an emergency, public health practitioners need to understand how to effectively use social media to rapidly disseminate information, so that the public health message goes viral,* instead of the disease. We propose a novel framework using a 3 Rs principle—Review, Recognize, and Respond—to help public health practitioners design tailored messages that prevent disease and promote health before, during, and after a public health emergency.

Social media has become a primary channel for information. Where news once was defined by traditional print and broadcast media, today's current events are more rapidly delivered and digested through devices at our fingertips. Younger Americans lead the trend for online news consumption—mainly through social media channels on mobile devices.^2^ According to the Pew Research Center,^3^ 36% of Americans 18 to 29 years of age consume their news through social media platforms, compared with news websites (27%), television (16%), radio (13%), and print (2%).

With approximately 6,000 tweets appearing on Twitter every second, the effect of social media on information sharing cannot be overstated.^4^ Public health practitioners should be acutely aware of the power of social media. The potential to expeditiously disseminate information, however, can be a double-edged sword. This is especially true during public health crises, since rapid messaging can either quickly ameliorate or harm a situation depending on how the message is received and interpreted.^5^ However, accurate and targeted social media messaging can promote health and save lives; its power should not be underestimated when measuring the impact on a population.^6,7^

The ongoing coronavirus disease 2019 (COVID-19) pandemic serves as a quintessential example.^8^ As the world grappled with the emergence of a pandemic, the global community also had to reckon with the rise of an *infodemic* as information was rapidly disseminated across various social media channels.^9^ The quick spread of information on social media can be measured by the R_0_, or reproduction number, for a social media platform analogous to an infectious pathogen's R_0_, which measures the spread of the pathogen.^10^ While many of these early messages were accurate and grounded in public health science, others did not align with official public health guidance but still gained traction.^11-13^ With emerging COVID-19 vaccines not widely available as of this writing, public health authorities needed to rely on public health messaging to promote nonpharmaceutical interventions (eg, use of cloth face coverings, hand hygiene, and physical distancing). Dissemination of public health messages is dependent on trusted and reliable media channels for outreach—both social media and traditional media—to prevent infections and protect populations.

Effective communication is critically important in disaster response as well.^14,15^ When Hurricane Florence lashed the Carolina coast in 2018, hashtags like #hurricaneflorence and #florence2018 garnered much attention and were trending upward on social media, but many tweets originated from unverified accounts or did not align with official public health guidance.^16^ As a result, public health messages became muddled in a slew of conflicting ideas and opinions. It is, therefore, important to establish identifiable accounts linked to official profiles on social media prior to an event/incident. For example, a case study analyzing tweets during hurricanes Harvey and Irma found that tweets from verified government accounts on Twitter were more likely to be retweeted and helped debunk rumors.^17^ The individuals associated with the accounts should readily maintain authentication information such as passwords to allow seamless account monitoring and use. For instance, there was a delay in correcting a false ballistic missile alert in Hawaii in 2018 by Governor David Ige because he could not immediately recall his Twitter password.^18^ Public health practitioners need to be savvy at social media messaging before, during, and after a public health emergency in order to ensure that the right message is sent out at the right time to save lives.^19^

Effective social media messaging can be developed if those messages are based on the principle of the 3 Rs. We propose this novel framework that public health practitioners can use to prepare for the next public health crisis, so that that the right social media messages—not the disease—go viral. This framework builds on other guides and toolkits, including the Centers for Disease Control and Prevention (CDC)'s social media toolkit for health communicators, in order to effectively communicate during a public health emergency.^20-23^

## 3 Rs: Review, Recognize, and Respond

**Figure 1.**
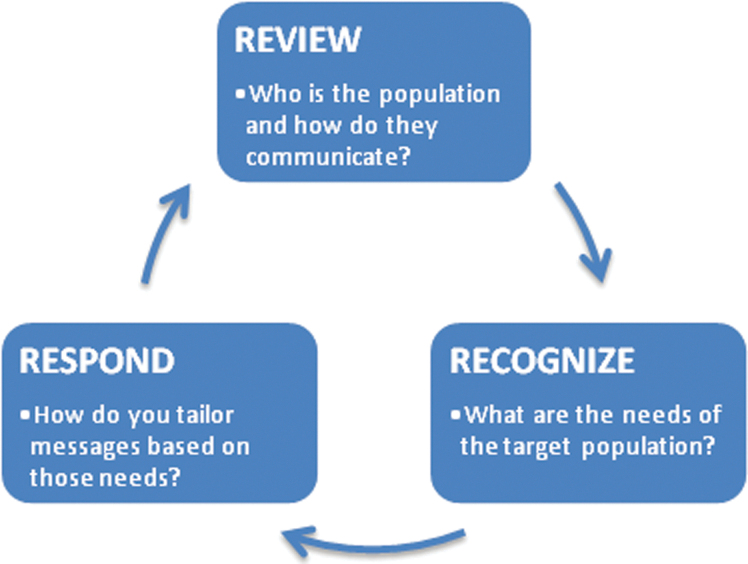
The 3 Rs of effective social media messaging during a public health emergency — Review, Recognize, and Respond.

The principle of the 3 Rs—Review, Recognize, and Respond—refers to the ongoing cyclical process of developing an effective social media communication strategy and evaluating the impact of that strategy (Figure 1). Designing effective public health social media messages is a skill that can be developed but requires consolidated and coordinated effort from public health practitioners and stakeholders. The 3 Rs is a systematic way to characterize the information needs of a target population and to ensure that social media messages are effectively meeting the needs of that population (Table 1).

**Table 1.** Implementation Guide for the 3 Rs of Effective Social Media Messaging During a Public Health Emergency

**The 3 Rs**	**Action Steps**
**Step 1: Review** the target audience	Prior to an emergency, conduct a needs assessment to: • Identify target population(s) in a community (eg, high-risk groups, sociodemographics) • Identify your specific target audience(s) (eg, 21-year-old Latina college student residing at a dormitory in McAllen, TX at high risk for meningococcal infection) • Identify the social network for the target audience(s) and the social media communication platforms they use (eg, Twitter, Facebook, Instagram, Snapchat, YouTube, TikTok) • Identify literacy levels of the target audience(s) (eg, health literacy, language literacy, digital and social media literacy) • Identify your followers and relevant influencers Develop generic message templates and test message effectiveness with the various target audiences (eg, clear messages with visual and interactive content tend to be more engaging). Ensure that your social media accounts are verified (eg, blue check on Twitter). Build trust and credibility.
**Step 2: Recognize** the health communication needs	Identify immediate needs of the target audience(s) in the immediate aftermath of the emergency. Conduct a rapid surveillance of social media to monitor and identify gaps and/or detect misinformation/disinformation on various communication platforms (eg, using social media analytics to understand the collective dialogue). Identify social media influencers who are shaping the communication (eg, celebrities, opinion leaders, organizations).
**Step 3: Respond** with tailored messages	Respond with customized messages for the various target audiences to meet the specific needs of the evolving crisis. Express empathy and continue to build and maintain trust and credibility. Continue to analyze social media metrics and dialogue to assess the message's impact and reach and further refine the tailored messages to manage the discourse and meet the needs of the population.

Note: The 3 Rs are not a mutually exclusive sequential process, but rather is an iterative process where 1 or more of these steps may occur in tandem. It is also important to continuously monitor and evaluate social media messaging using various analytic tools to keep up with the rapid change in the landscape and to apply lessons learned in near-real time.

### Step 1: Review Target Audience

The first step—Review—refers to the preevent surveillance process, where public health risk communicators review a target population to understand how to best communicate information before a disaster. At its most fundamental level, the Review process involves identifying the literacy levels of a target population, including health literacy (the ability to obtain, process, and understand to make informed health decisions), language literacy (reading and writing ability), and digital and social media literacy. During an emergency, messages need to be clear, succinct, jargon-free, and tailored to the literacy level of the target audience. Risk communicators should not waste valuable time during an emergency to understand a population's literacy levels; this information should be included in preparedness plans where population demographics are well-defined. The 2014-2016 West Africa Ebola outbreak is a good case example illustrating how risk communicators designed messages aligned with the literacy levels of a target population. The countries affected by the epidemic spanned multiple sociocultural groups, each with their own language, dialect, and varying levels of literacy. To ensure rapid dissemination of information, risk communicators relied on pictorial representations to convey health messages, since text-heavy documents may not have been easily understood, given the culturally and linguistically diverse landscape.^24^

In addition to reviewing the literacy level of a target population, the Review process also involves understanding the “infrastructure” of social media communication channels before a public health emergency. Communication planning should address questions like: Which social media platforms are most popular in a target population? How do you curate and package social media messages in a way that skillfully uses a platform's algorithm so that the message is more likely to go viral? How does a trending message affect communal behavior or influence the collective psyche? Insights into an audience's social media preferences and practices can inform how culturally appropriate messages are designed to closely align existing social media practices.

Audience insight also involves understanding the sociodemographic characteristics and the social networks of a community. By identifying these characteristics of the target audience, messages can be drafted to ensure content resonates with community consumers.^25^ Before a crisis strikes, public health practitioners should conduct needs assessments to better understand the community's knowledge, attitudes, values, and practices about receiving and interpreting information. Additionally, public health practitioners at the local level should have a strong understanding of the specific social networks of their local community and, ideally, of subgroups within that community. For example, although Facebook and YouTube are the most common platforms for most Americans younger than 65 years of age, young adults (ages 18 to 24 years) are more likely to use Snapchat, Instagram, and Twitter, while adolescents tend to prefer TikTok.^26,27^ Risk communicators aware of this preference are better positioned to provide meaningful messaging for different social media platforms, especially when targeting certain sociodemographic groups.

In the Review stage, risk communicators need to establish a strong social media presence to gain and maintain followers before the event or incident occurs. Followers can range from public health and community organizations, professional societies, and activists and influencers, in addition to members of the public at large. Furthermore, public health practitioners should maintain a verified account (eg, having a blue check on Twitter) to indicate that they are an official organization with public health authority in their jurisdiction. Before the event, risk communicators can test messages based on audience insight and literacy levels to see (1) which messages tend to go viral and (2) how to effectively tailor messages to capture an audience's attention, especially by using appropriate hashtags that resonate with target audiences.^28^ Public health's “voice” needs to be established as a credible resource on social media before a disaster strikes so that when a crisis does occur, individuals will naturally turn to public health's social media presence for guidance.

### Step 2: Recognize Health Communication Needs

Disasters are abrupt disruptions to routine daily activities that often result in personal injury, property damage, and frequently have profound environmental and economic effects. The sudden nature of most public health emergencies can deeply affect the psychological health of populations, leaving individuals searching for information to protect life and property. In the immediate aftermath of a disaster, public health risk communicators need to rapidly transition from the first step, Review, to the second step, Recognize, in order to effectively meet the informational needs of affected populations.

The second step, Recognize, encompasses the process of rapid surveillance of communication streams to identify gaps or misinformation and disinformation in the collective social dialogue. Misinformation refers to unintentional distortion of content through error or ignorance, while disinformation refers to an intentional distortion of content with the intent to deceive. Social media analytics can facilitate understanding about message uptake by various audiences and who is amplifying messages (influencers).^29,30^ If social media influencers are shaping the conversation, consider whether they can be leveraged to share appropriate risk communication. Social media influencers such as celebrities, opinion leaders, and experts have the potential to propagate and amplify a message. For example, comedian Chris Rock tweeted to his 5.2 million Twitter followers emphasizing the need to social distance during the COVID-19 pandemic.^31^

Another critical component of the Recognize step is to identify whether an organization or a stakeholder is sending out appropriate public health information, or worse, misinformation. Is there a threat to public health's credibility or widespread doubt about the way the authorities are handling the response? In the era of social media where informational flow is organic and bidirectional, risk communicators need to monitor the constant flux of information in order to assess the tone of the collective discourse and to provide guidance at critical times. This was highlighted during the public health response to the COVID-19 pandemic, when guidance was often questioned and public health messaging became politicized.^32^ Evaluating sources, intentions, and platforms for misinformation in real-time can inform the development of effective interventions that target an infodemic during a public health crisis. It is important that risk communicators frequently check the pulse on multiple social media channels to quickly recognize the issues as they arise during this second step.

Populations facing unprecedented crises often have very specific informational demands that public health practitioners need to identify. In the aftermath of an errant emergency alert of a ballistic missile threat in Hawaii in January 2018, a qualitative analysis of posts on Twitter identified several emerging themes, highlighting how the public seeks information at different points in time. The initial themes that were identified in the immediate aftermath of the emergency alert included informational processing, informational sharing, authentication, and emotional reactions. Themes that emerged after the missile warning was redacted included denunciation, insufficient knowledge to act, and mistrust of authority.^33^ Risk communicators should be aware of themes as they emerge during an unfolding crisis to make sure that the right message is sent at the right time to address the public's need.

In today's digital age, social media is often the first publicly available information when an incident occurs. Rapidly recognizing the informational needs of the public is a crucial first step before risk communicators design and disseminate messages to mitigate the impact of disasters and save lives.

### Step 3: Respond with Tailored Messages

The final step in the principle of the 3 Rs—Respond—is a natural consequence of the second step, Recognize. Once risk communicators recognize gaps and/or misinformation or disinformation on social media channels, they need to respond with tailored messages that address and mediate that gap. Public health practitioners can provide real-time information, including clear instructions for steps to take during an emergency, while expressing empathy, building trust, and establishing credibility.^34^ Developing the trust of institutions and organizations as well as of public officials and leaders is crucial for messages to have credibility among lay audiences.^35^

To prepare for the Respond step, best practices for risk communicators suggest developing generic message templates, facts sheets, and resource pages for various scenarios likely to occur in a particular jurisdiction or that may affect a population. These messages are often short, as dictated by the limits of the various social media platforms (eg, 280 character limit for Twitter). Planning templates can readily be tweaked during a crisis and disseminated rapidly, whereas developing messages organically during a crisis can be time-consuming and daunting since emotions often run high and people process information differently and potentially lead to communication errors.^36^ Predeveloped messages designed to communicate within the character limits of the various social media platforms, can be rapidly reviewed and updated to meet the specific needs of the evolving crisis. It is also important to have a plan for correcting any inaccuracies that may be inadvertently disseminated.

Social media messages need to be clear, engaging, and coordinated across various social media platforms. Depending on the health issue, use visual content messages (eg, infographics, photos, videos, gifs, memes, quote graphics) as well as interactive content (eg, polls, quizzes) that is “witty” and “catchy” in order to engage, inform, and encourage public health action. Clearly, the tone of any messaging should align with the health issue or crisis (eg, humor or wit may not be appropriate in many circumstances). Multiple studies from various disciplines confirm the old adage that a picture is worth a thousand words, and suggest that images are more memorable and effective than text alone.^37,38^ Market research also confirms that visual content generates more views and are more likely to be shared.^39,40^ Fact sheets, public service announcements, and videos can be used to augment the health communication message. Chats on social media platforms (such as Twitter and Facebook) can be used to further engage with the target audience.

Finally, social media posts need to be genuine and transparent. Public health practitioners may not have all the answers in the immediate aftermath of a disaster or during the initial stages of a public health emergency. Public approval is often higher when officials offer assurances about what is confirmed and delineate areas that are still under investigation.^19^ A genuine, transparent, and honest response builds credibility with the target audience. A message that appears disingenuous can instantaneously backfire and create backlash.

The bidirectional nature of informational flow on social media allows public health practitioners to not only respond to informational gaps or misinformation on social media channels, but also allows for the public to respond and comment in real time. This bidirectional process enhances transparency, mediates rumors, and actively informs an engaged population during an emergency. As the public health message accelerates and goes viral, risk communicators can review how the public is responding and reacting to that message, thereby starting the cycle of the 3 Rs once again.

In summary, the process of developing effective social media messages must begin before a disaster occurs, and the Review step encompasses all these preevent activities. By learning a population's literacy level, understanding the social media infrastructure, and by building followers on social media channels, public health risk communicators will be ready to respond during a public health emergency. Furthermore, by strengthening the social media infrastructure, public health officials will be more prepared to recognize their community needs and respond rapidly with tailored messages on appropriate social media platforms to adequately meet the needs of their population.

## Discussion

The 3 Rs of social media is a framework that provides a systematic way to think through the multifaceted steps involved in generating an effective social media message. Public health practitioners and risk communicators can utilize this framework to gain better insight about their target audience, effectively reach large segments of their jurisdiction, and provide real-time information during an evolving crisis. The 2-way communication between the public and public health practitioners allows for rapidly distributing lifesaving messages while addressing public concerns and panic during an emergency.

New research shows that the viral spread of information can be compared to an infectious pathogen's R_0_, where information shared on social media platforms such as Twitter, Instagram and YouTube can have R_0_ > 1 indicating an infodemic, analogous to an epidemic.^10^ Segmented media markets are creating opportunities for the spread of misinformation and disinformation that could lead to confusion.^41^ As social media channels diversify to meet niche audiences, the risk of fragmented messages spreading rapidly across social media platforms increases.^42^ It is, therefore, imperative for public health practitioners to monitor the social media landscape and stay ahead of the “info-curve” before information is misrepresented and goes viral.^43^ Recently, social media platforms such as Twitter and Facebook tried to combat misinformation and disinformation by flagging posts they determine to be misleading.^44^ Analyzing how social media content is shared and consumed across diverse platforms can help inform targeted risk communication that is actionable and effective.

Furthermore, it is important to note that the 3 Rs are not a mutually exclusive sequential process, but rather is a cyclical process where 1 or more of these steps occurs in tandem. For instance, if a mass inhalational anthrax exposure were to occur in a major metropolitan area, it is public health's imperative to respond quickly. Waiting in the Recognize step to identify knowledge gaps and/or misinformation would not be prudent when some Response messages can be developed immediately. Nevertheless, once an initial message is disseminated, public health practitioners should engage in the Recognize and Review steps to manage the discourse on social media.

As in so many other areas of public health, continuous evaluation and monitoring remains central to social media messaging. Throughout all steps of the 3 Rs, public health practitioners need to reassess social media metrics—such as retweets, quote tweets, likes, shares, and impressions—to determine the message's impact and reach. The power of social media lies in its ability to inform and engage communities, and public health practitioners need to be aware of this potential. By monitoring metrics as an event/incident evolves, risk communicators can accurately track how a message is shaping social dialogue and influencing lifesaving actions. Furthermore, monitoring and evaluation will also allow public health practitioners to keep up with rapid change in the world of communication on social media and to apply lessons learned in near real time. It is important to continually assess the barriers and facilitators of social media messaging in an ever-evolving landscape with new communication technologies in order to maximize strategic efforts to reach various target groups in a community.^45^ Effective communication is essential for public health preparedness, response, and recovery, and is a core function of Emergency Support Function 8 (ESF8)^46^ and the CDC Public Health Emergency Preparedness (PHEP)^47^ capabilities. Developing a communication strategy that effectively harnesses the power of social media not only aligns with core functions of public health but also ensures that that the right message goes viral during the next public health emergency.

## Notes

^*^
Going viral is defined as an explosive dissemination of a message analogous to how viruses replicate.
